# Editorial: Critical perspectives on gender equality policies and practices for staff in higher education

**DOI:** 10.3389/fsoc.2022.984724

**Published:** 2022-08-02

**Authors:** Gail Crimmins, Sarah Barnard

**Affiliations:** ^1^University of the Sunshine Coast, Maroochydore, QLD, Australia; ^2^Loughborough University, Loughborough, United Kingdom

**Keywords:** gender equality, gender equality plans (GEP), resistance, gender bias, higher education, tertiary education

This Research Topic shares critical perspectives on gender equality policies and practices for staff across the international higher education. There is evidence of persistent and entrenched gender inequity in the staffing of universities and research centers. On average women represent between 23 and 57 per cent of academic roles in higher education in all OECD countries with available data, most below the 50 per cent level (OECD, 2019). At senior levels, women make up 22 per cent of heads of all Higher Education Institutions (HEIs) and 14 per cent of the heads of universities across the EU (European Commission, 2019). Similarly, 39 out of the top 200 institutions in the world (19.5 per cent) are currently led by women (Bothwell, 2020), and when women lead institutions, they are disproportionately more likely to be smaller colleges or women's universities, particularly in South Asian countries (Morley and Crossouard, 2016). Correspondingly, in Japan, South Korea, and Hong Kong, the top national or public universities that have entered the highest ranks of the international league tables are all led by male presidents (Cheung, 2021). Therefore, despite some variance in proportions of women in academic roles across the globe, higher education institutions remain indisputably gendered organizations (Acker, 2006).

Dominant rationales for addressing this phenomenon include gender inequity in the academy wastes female talent (Blackmore, 2014) that leads to an underperformance of research capacity and poor return on financial investment and human resources (Henderson and Herring, 2013)—a business case for equality. In addition, it is posited that universities have a moral mandate to ensure women are properly represented in senior academic positions in universities to help female students envision themselves in leadership roles in the organizations into which they will enter as graduands. Finally, gender equity is presented as central to social and epistemic justice (Clavero and Galligan, 2021).

Importantly, funding bodies have used research funding mechanisms as a lever for change, embedding consideration and actions on gender inequality into funding applications processes. For instance, the European Commission has mandated that institutions applying to the Horizon Europe research and development programme have GEPs in place (European Commission, 2021); and the body that convenes the research Councils in UK (UKRI), though not requiring organizations to secure equity awards to access funding, it has stated that it expects those in receipt of Research Council funding to embed equality and diversity in aspects of research practice (UKRI, 2022).

Consequently, many research and higher education institutions are implementing gender mainstreaming and gender equality plans (GEPs) as vehicles to support gender equity. There are as many as 113 gender and diversity Certification and Award schemes (CAS) identified across Europe and beyond (Nason and Sangiuliano, 2020). Therefore, in many contexts, discourses informing policy frameworks have shifted from equal opportunities and gender equity as a social justice imperative, to a “managing diversity” focus that is promoted as better for business and in the national economic interest; and from widespread acceptance of societal support and collectivist alliance to individualism and responsibilisation (Crimmins, 2021). Finally, because COVID-19 has negatively impacted most counties economies, and cuts in gender equality structures occur during times of economic downturn and corresponding austerity (Briskin, 2014), as editors of the Research Topic, *Critical perspectives on gender equality policies and practices for staff in higher education*, we sought to understand the current context and efficacy of gender equity plans and policy within higher education. We therefore invited research and position papers that discuss effective gender equity policy, current gender equity policy maneuvers, and the impact of COVID-19 on gender equity policy frameworks.

Eight papers were published in the series, representing insights from 8 countries (3 in the Global South and 5 in the Global North) and 43 academics (29 of whom are based in Brazil, 4 in the US, 3 in the UK, 2 in Australia, 2 in Ireland, 1 in Austria, 1 in Germany, 1 in Sweden). These papers underwent double blind review by 16 generous reviewers (5 based in the UK, 2 in Germany, 2 in Spain, 2 in the US, 2 in Australia, 1 in Italy, 1 in New Zealand, 1 in South Africa). We acknowledge that the series unintentionally draws on expertise on the critical analysis of gender equality work within and through the lens of the Global North. This reflects two phenomena: First, that formal gender equality policies are most commonly employed (and generally named as such) across the Global North; and second that academics from these regions feel most prepared in reviewing academic papers that focus on this topic.

The methodologies and methods employed across the 8 papers include: a literature review outlining what we know about implicit bias in academia (also known as unconscious bias), with recommendations for action from the perspective of a group of Latinx American scientists comprising Black and Latina women, teachers, and undergraduate students who participate in women in science working group at universities in the state of Rio de Janeiro, Brazil (Calaza et al.); analysis of a national survey in which US professors (*n* = 364) responded to vignettes of three hypothetical undergraduates, rating the extent to which they would encourage male and female students to pursue a Ph.D. in physics regardless of whether this course of action matches the students goals and interests (Bailey et al.); a qualitative questionnaire methodology used to garner written narratives based on the lived experience of women working in an Australian regional university (White and Goriss-Hunter); an analysis of the influence of gender, parenthood, and race on academic productivity during the pandemic period based on a survey of Brazilian academics (*n* = 3,345) from various knowledge areas and research institutions (Staniscuaski et al.); a case study employing thematic content analysis of institutional documents pertaining to gender equality, with a focus on internal promotions to Associate Professor in an Irish university (Hodgins and O'Connor); an action research-based ethnographic methodology used to explore practices “change agents” experienced as useful and important for promoting gender equality in their different organizational contexts (Dahmen-Adkins and Peterson); a comparison of two of the main gender equality schemes used by research-performing organizations in Europe based on qualitative interviews with stakeholders and document analysis (Tzanakou et al.); and a critical evaluation of the Austrian quota regulation and the gender competence policy in Austria, including implementation and limitations of these approaches (Wroblewski). The methodological approaches were somewhat varied, including both qualitative and quantitative methods. This has resulted in useful overviews of the situation in different contexts, in-depth consideration of policy documentation and the gathering of rich data as a women share their stories. The Research Topic, therefore, provides a comprehensive assessment of the various elements of academic productivity relevant to a wide range of knowledge areas and research institutions.

Whilst the papers published in this Research Topic provided varied new knowledge and insights, there were two main preoccupations that permeate most of the papers published: First, an endurance of discrimination that is (re)expressed at a cultural level with institutions acting as sites of resistance in the face of pressure to change; and second, the impact of gender intersections with race on inequalities in the higher education sector. Whilst the intentions of gender equality policy and practices were generally described as positive and having achieved some success, gender bias and resistance to gender equality actions were presented as difficult to explicitly discern and disrupt. Although potential solutions and strategies for effective change practices were also focused upon. The authors of these papers proffer specific, actionable strategies and interventions to address these issues, and future research based on the implementation and assessment of the suggested strategies is recommended. A further recommendation is for collaborations gender equality policy and practice mapping and implementation between scholars located across the Global North and South, to expand discourses around gender equity within the international higher education sector.

## Author contributions

GC and SB contributed to this work and have approved it for publication.

## Conflict of interest

The authors declare that the research was conducted in the absence of any commercial or financial relationships that could be construed as a potential conflict of interest.

## Publisher's note

All claims expressed in this article are solely those of the authors and do not necessarily represent those of their affiliated organizations, or those of the publisher, the editors and the reviewers. Any product that may be evaluated in this article, or claim that may be made by its manufacturer, is not guaranteed or endorsed by the publisher.
